# *Dendropanax trifidus* Sap-Mediated Suppression of Obese Mouse Body Weight and the Metabolic Changes Related with Estrogen Receptor Alpha and AMPK-ACC Pathways in Muscle Cells

**DOI:** 10.3390/nu14051098

**Published:** 2022-03-05

**Authors:** Ahreum Lee, Eugene Koh, Dalnim Kim, Namkyu Lee, Soo Min Cho, Young Joo Lee, Ik-Hyun Cho, Hyun-Jeong Yang

**Affiliations:** 1 Korea Institute of Brain Science, Seoul 06022, Korea; dkfma5025@hanmail.net (A.L.); hoipig0326@naver.com (D.K.); 2 Temasek Life Sciences Laboratories, Singapore 117604, Singapore; eugene@tll.org.sg; 3 Department of Integrated Bioscience and Biotechnology, College of Life Science, Sejong University, Seoul 05006, Korea; nam879@naver.com (N.L.); yjlee@sejong.ac.kr (Y.J.L.); 4 PharmCADD, R&D Center, Seoul 06180, Korea; chosm@pharmcadd.com; 5 College of Korean Medicine, Kyung Hee University, Seoul 02447, Korea; ihcho@khu.ac.kr; 6 Department of Integrative Health Care, University of Brain Education, Cheonan 31228, Korea; 7 Department of Integrative Biosciences, University of Brain Education, Cheonan 31228, Korea

**Keywords:** *Dendropanax trifidus*, body weight, C2C12, metabolism, glycolysis, mitochondrial respiration, AMPK, ACC, estrogen

## Abstract

*Dendropanax trifidus* (DT) is a medicinal herb native to East Asia, which has been used extensively for its therapeutic properties in traditional medicine. In this study, we examined the effects of DT sap on the regulation of body weight and muscle metabolism in mice. Obese model db/db mice were administered daily with DT sap or vehicle control over a 6-week period. The effects of DT sap on muscle metabolism were studied in C2C12 muscle cells, where glycolytic and mitochondrial respiration rates were monitored. As AMP-activated protein kinase (AMPK) is a master regulator of metabolism and plays an important function as an energy sensor in muscle tissue, signaling pathways related with AMPK were also examined. We found that DT sap inhibited body weight increase in db/db, db/+, and +/+ mice over a 6-week period, while DT sap-treated muscle cells showed increased muscle metabolism and also increased phosphorylation of AMPK and Acetyl-CoA Carboxylase (ACC). Finally, we found that DT sap, which is enriched in estrogen in our previous study, significantly activates estrogen alpha receptor in a concentration-dependent manner, which can drive the activation of AMPK signaling and may be related to the muscle metabolism and weight changes observed here.

## 1. Introduction

Decrease of energy expenditure can increase body weight [1,2]. In other words, a person with lower metabolic rate gains more weight under the same caloric intake. The skeletal muscle is the largest metabolic organ system in the body [3]. It comprises ~40% of body weight in non-obese individuals [4] and is responsible for 20~30% of total resting oxygen uptake [4,5]. Skeletal muscle metabolism is an important determinant for whole-body resting metabolic rate [6], and changes dramatically depending on exercise activity.

During exercise, skeletal muscle ATP consumption increases, followed by increase in intracellular AMP concentrations, resulting in increases in the ratios of AMP/ATP and ADP/ATP, and leading to the activation of AMP-activated protein kinase (AMPK) [7]. AMPK is a sensor for intracellular energy status which maintains energy storage by fine-tuning anabolic and catabolic pathways, and is especially important for skeletal muscle, which experiences rapid energy turnover [8]. In order to maintain intracellular energy homeostasis, AMPK regulates a broad range of intracellular downstream signaling molecules such as Acetyl-CoA Carboxylase (ACC) and 6-phosphofructo-2-kinase/fructose-2,6-biphosphatase3 (PFKFB3). ACC, which catalyzes the carboxylation of acetyl-CoA to malonyl-CoA [9], can be phosphorylated by AMPK, resulting in inhibition of its enzymatic activity [10]. ACC has been previously investigated as a potential target of anti-obesity drugs [11,12]. PFKFB3 is a glycolytic enzyme which plays roles in glycolysis, cell proliferation, and tumor growth, and its selective inhibition is potentially regarded as a therapeutic target [13]. Likewise, AMPK is regarded as a central regulator for energy homeostasis, thus compounds which activate AMPK become targets for drug development [14]. Intriguingly, like exercise, estrogen also increases AMPK phosphorylation [15,16]. Estrogen plays a critical role in skeletal muscle homeostasis and exercise capacity [17] and can improve exercise endurance and mitochondrial energy metabolism in mice [17].

*Dendropanax trifidus* (DT), phylogenetically similar to *Dendropanax morbiferus* (DM), is an evergreen shrub which is mainly distributed throughout eastern Asia (including Korea and Japan) and has been appreciated for its therapeutic properties in Korean traditional medicine. DM and DT are hard to distinguish due to their close genetical and morphological characters [18,19]. In this aspect, Lee and colleagues [20] claimed that DT and DM should be considered as conspecific under *D. trifidus*. DM leaf extract was found to inhibit adipogenesis in mouse 3T3-L1 cells [21], and reduced body weight in high fat diet-fed C57BL/6 mice [22]. (9Z,16S)-16-hydroxy-9, 17-octadecadiene-12, 14-diynoic acid (HOD), a component of DM leaf, reduced oleic acid-induced TG accumulation in HepG2 hepatocytes, contributing to the reduction of body weight [23]. Recently, in a 12-week RCT, participants administered with DT leaf extract exhibited significant reductions in HbA1c, insulin resistance level and systolic BP, compared to the participants with placebo, however, lipid parameters and body composition including body weight were not changed [24]. Compared to the studies with DM leaf extract, not much is known about the effects of DM or DT sap.

In this study, we aimed to understand the effects of DT sap on mouse body weight and muscle metabolism. We administered DT sap via oral injection over a 6-week period to obese (db/db), heterozygous (db/+), and control (+/+) mice and measured their body weight at weekly time points. We further examined the effects of DT sap on glycolysis and mitochondrial respiration parameters in C2C12 skeletal muscle cells. To study the underlying molecular mechanism, we examined whether the function of DT sap is mediated by estrogen receptor, as estrogen was one of the major components of DT sap [25]. Finally, we show here AMPK and its downstream signaling—including ACC and PFKFB3 which are significantly altered by DT sap treatment, implying that those cellular components might mediate glycolytic and metabolic changes within the cell, thus contributing to weight loss in mice treated with DT sap.

## 2. Materials and Methods

### 2.1. Mice

**Figure 1:**
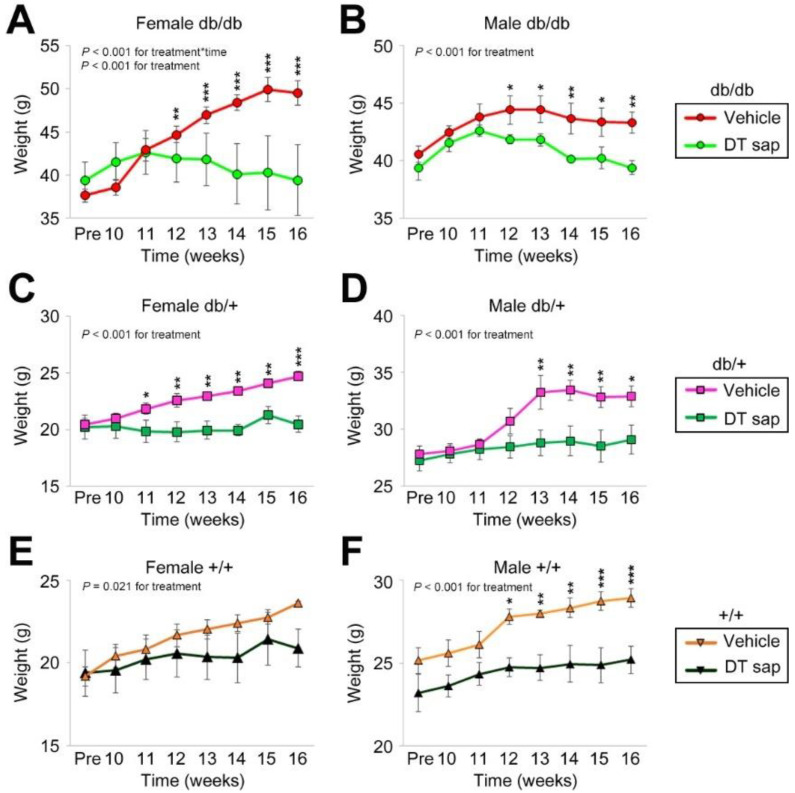
Body weight change by *Dendropanax trifidus* sap compared to vehicle administration in db/db, db/+, +/+ mice for 6 weeks. 0.2 mg/g DT sap or vehicle control (100 µL) was administered daily for 6 weeks to db/db (**A**,**B**), db/+ (**C**,**D**), and +/+ (**E**,**F**) mice (female: (**A**,**C**,**E)**; male: (**B**,**D**,**F**)). Body weights were measured every week. Data was analyzed by two-way ANOVA. N = 3–10 mice per group. * *p* < 0.05, ** *p* < 0.01, *** *p* < 0.001. Bars indicate mean ± S.E.M.

All experiments were performed in compliance with the relevant laws and institutional guidelines and were approved by the University of Brain Education’s Animal Care and Use Committee (Approval number: 2018-AE-01Y-01). For obesity model mice, BKS.Cg-*Dock7^m^* +/+ *Lepr^db^*/J lines, which are used for mouse model for type II diabetes and obesity, were purchased from ORIENT BIO Inc. (Seongnam, Korea). Homozygous mice (db/db) manifest morbid obesity. Ten-week old female and male mice with genotype of db/db, db/+, +/+ (3~10 mice per genotype per sex) were administered daily with 100 μL (per mouse) of vehicle or DT sap (0.2 mg/g for DT sap weight/mouse body weight) via oral gavage for six weeks. Body weights were measured every week (Figure 1).

### 2.2. Reagents

An estrogen receptor antagonist ICI 182,780 (Fulvestranat, 14409) and β-estradiol (E2758) was purchased from Sigma-Aldrich (Saint Louis, MO, USA). *Dendropanax trifidus* (Thunb.) Makino ex H.Hara from Simasi, Mieken, Japan was registered at Ibaraki Nature Museum (INM-2-212778). *Dendropanax trifidus* sap was ethanol-extracted, freeze-dried and stored at −20 °C. For experiments, the freeze-dried final product was dissolved in EtOH and diluted with water or medium [25].

### 2.3. Antibodies

Rabbit polyclonal or monoclonal antibodies against phospho-AMPKα (Thr172) (#2531), AMPK (#2532), phospho-HSL (#4137T), HSL (#4107T), phospho-ACC (Ser79) (#11818T), ACC (#3676T), ATGL (#2138S), phospho-ULK1(Ser555) (#5869T), and ULK1 (#8054T) were purchased from Cell Signaling. Rabbit polyclonal antibodies against PFKFB3 (#13763-1-AP) and b-Actin (#ab8227) were purchased from Proteintech (Rosemont, IL, USA) and Abcam (Cambridge, UK), respectively.

### 2.4. C2C12 Cell Culture

Murine myoblast cell line C2C12 cells were routinely cultured in growth medium containing 10% heat-inactivated fetal bovine serum (FBS)/1% penicillin streptomycin (PS)/Dulbecco’s Modified Eagle’s Medium (DMEM) without phenol red. Cells were cultured in chamber-slides at 37 °C in a humid atmosphere of 5% CO_2_ in air. Cultures were passaged every 2 days with fresh medium. For differentiation, when cells reached confluence (at the 3rd day after seeding with a density of 1 × 10^4^ cells/well for the 6-well plate), the medium was changed into 2% horse serum/1% PS/DMEM without phenol red. After three days of differentiation when cells were fused into myotubes, cells were treated with the indicated reagents for the indicated time duration and analyzed by Western Blot analysis.

### 2.5. Western Blot

Cells were lysed in cold RIPA buffer (WSE-7420, ATTO, DAWINBIO Inc, Hanam, Korea) on ice and collected for centrifugation at 15,000 RPM for 15 min. The resultant supernatant was quantified and diluted with sample buffer, boiled, subjected to SDS-PAGE, and transferred onto PVDF membranes. The membranes were blocked with EZBlock Chemi (AE-1475, ATTO, Tokyo, Japan) for 1 h at room temperature, incubated with the primary antibodies overnight at 4 °C, washed and further incubated with secondary antibody for 1 h at room temperature, and washed again. Protein signals were visualized by Super Signal West Pico PLUS Chemiluminescent Substrate (34580, Thermo Fisher Scientific, Waltham, MA, USA) and images were taken by Amersham Imager 600 (GE Healthcare, Chicago, IL, USA). Western blot images were quantified by Image J software (version 1.52p, NIH, Bethesda, USA).

### 2.6. Luciferase Assay and Cell Viability Test

For luciferase assay, the hERα-HeLa-9903 cell line expressing hERα was used. Cells were maintained in 10% FBS/DMEM. On the day of the experiment, cells were seeded in 10% DCC/FBS/DMEM on 96 well plates with a density of 1 × 10^4^ cells/100 μL/well, and maintained at 37 °C in a 5% CO_2_ incubator for 24 h. The cells were treated as indicated and further incubated for 48 h at 37 °C in a CO_2_ incubator. The medium was removed and luciferase activity was measured by luminometer.

Cell viability check was used by Cell proliferation reagent (Thiazolyl Blue Tetrazolium Bromide) (Sigma, St Louis, MO, USA) for measurement at the absorption at 590 and 620 nm of wavelength by absorbance microplate reader (Bio-Tek, Winooski, VT, USA).

### 2.7. Glycolysis, Mitochondrial Stress Test, and Energy Map

C2C12 myoblast cells were seeded with a density of 0.5 × 10^4^ cells/well on 96-well plates for the Agilent Seahorse XF assay, differentiated under the differentiation medium (2% horse serum/1% PS/DMEM) for three days, then treated with DT sap of the indicated concentrations (0, 62.5, 125, 250, 500 μg/mL) for 1 h or 24 h, and subjected to Glycolysis or Mitochondrial stress tests according to the manufacturer’s instructions (Agilent Seahorse XF Glycolysis Stress Test Kit 103020-100, Agilent Seahorse XF Cell Mito Stress Test Kit 103015-100, Agilent Technologies, Santa Clara, CA, USA). Each treatment was performed in triplicate and independent experiments were performed in duplicate.

**Figure 2:**
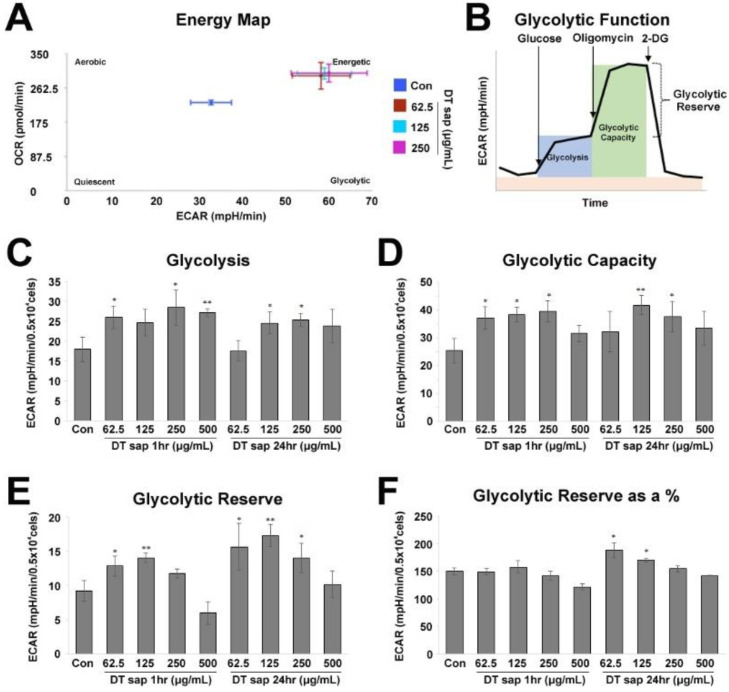
Glycolysis stress test for *Dendropanax trifidus* (DT) sap in C2C12 cells. (**A**) Energy phenotype profile of control cells (blue) and cells treated with DT sap of the indicated concentrations for 1 h (red, 62.5 μg/mL; light blue, 125 μg/mL; purple, 250 μg/mL). (**B**–**F**) Glycolysis Stress test for measuring glycolytic function in cells by direct measurement of the extracellular acidification rate (ECAR). (**B**) Agilent Seahorse XF Glycolysis Stress Test profile. Indicated compounds (i.e., 10 mM glucose, 1 μM oligomycin, and 50 mM 2-deoxy-glucose) were sequentially injected. Analysis details (i.e., Glycolysis, Glycolytic Capacity, and Glycolytic Reserve) are indicated. Image modified from Agilent Seahorse XF User Guide of the Test Kit. (**C**–**F**) Glycolysis stress test profiles are investigated by measuring ECAR from the cells of control or DT sap-treatment for 1 or 24 h. (**C**) Glycolysis. (**D**) Glycolytic capacity. (**E**) Glycolytic reserve. (**F**) Glycolytic reserve as a %. Con, Control; DT, *Dendropanax trifidus*; ECAR, extracellular acidification rate; OCR, oxygen consumption rate. N = 3. Student’s *t*-test, comparison with the control: * *p* < 0.05; ** *p* < 0.01. Bars indicate mean ± S.D.

Agilent Seahorse XF technology provides a cell energy metabolism map (a cell energy phenotype profile, Figure 2A) by a simultaneous measurement of two major energy production pathways, i.e., mitochondrial respiration and glycolysis in live cells. Cells are distributed in the energy map according to the following attributes: Quiescent (not very energetic via either metabolic pathway), Energetic (utilizing both metabolic pathways), Aerobic (utilizing predominantly mitochondrial respiration), and Glycolytic (utilizing predominantly glycolysis).

For the Glycolysis Stress Test, 10 mM glucose, 1 μM oligomycin, and 50 mM 2-deoxy-glucose (2-DG) were applied according to the manufacturer’s instructions (Figure 2B). Cells were incubated in the glycolysis stress test medium without glucose or pyruvate, then a saturating concentration of glucose was injected, inducing cells to catabolize it through the glycolytic pathway to pyruvate, producing ATP, NADH, water, and protons. The glucose-induced extrusion of protons into the surrounding medium rapidly increases extracellular acidification rate (ECAR), which is reported as the rate of glycolysis under basal conditions (Figure 2B, blue region). Glycolytic capacity is the maximum ECAR rate reached by a cell following the addition of oligomycin, which shuts down oxidative phosphorylation and drives the cell to reach its maximum glycolytic capacity (Figure 2B, green region). Glycolytic reserve indicates the capability of a cell to respond to an energetic demand and is calculated by the subtraction of the values of Glycolysis from Glycolytic Capacity (Figure 2B). Glycolytic reserve as a % is a calculation of (Glycolytic Capacity Rate)/(Glycolysis) x 100 (Figure 2B)).

**Figure 3:**
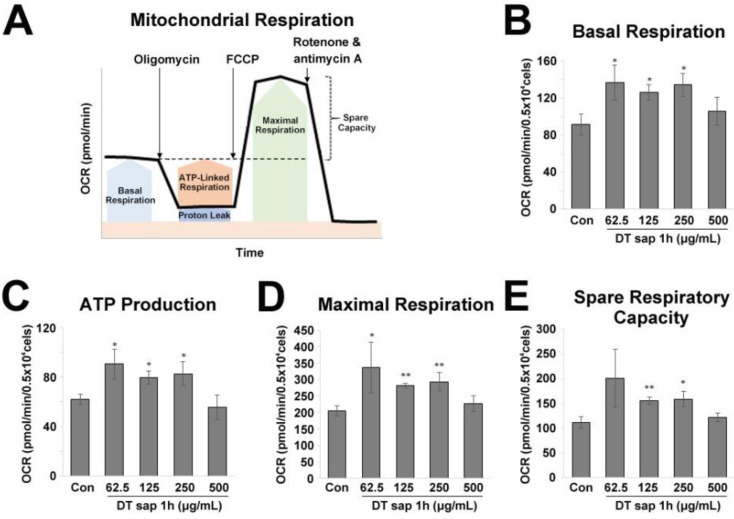
Mitochondrial stress test for *Dendropanax trifidus* (DT) sap in C2C12 muscle cells. (**A**) Agilent Seahorse XF Cell Mito Stress Test profile. Indicated compounds (i.e., 1 μM oligomycin, 1 μM FCCP, and 0.5 μM rotenone + 0.5 μM antimycin (**A**) were sequentially injected. Image modified from Agilent Seahorse XF User Guide of the Test Kit. (B–E) The oxygen consumption rate of C2C12 muscle cells was monitored at 1 h since the treatment of various concentrations of DT sap (0, 62.5, 123, 250, 500 µg/mL). (**B**) Basal respiration rate. (**C**) ATP production. (**D**) Maximal respiration. (**E**) Spare respiratory capacity. Con, Control; DT, *Dendropanax trifidus*; OCR, oxygen consumption rate. N = 3. Student’s *t*-test, * *p* < 0.05, ** *p* < 0.01, comparison with vehicle control. Bars indicate mean ± S.D.

For the Mito Stress Test, 1 μM oligomycin, 1 μM FCCP, and 0.5 μM rotenone + 0.5 μM antimycin A were applied in order (Figure 3A). In the mito stress test, basal respiration, ATP production, maximal respiration, and spare respiratory capacity were measured. Basal respiration indicates energetic demand of the cell under baseline conditions. ATP production by mitochondria is measured by the reduced OCR upon the addition of ATP synthase inhibitor oligomycin. Maximal respiration is measured by the increased OCR upon the addition of the uncoupler FCCP, which induces rapid oxidation of substrates to stimulate the respiratory chain to operate at maximum capacity. Spare respiratory capacity is calculated by subtracting basal respiration from maximal respiration and indicates the cell’s ability to respond to an energetic demand.

### 2.8. Statistics

Two-way ANOVA and post hoc Holm–Sidak analysis were used for body weight analysis. Student’s *t*-tests were used for other analyses.

## 3. Results

### 3.1. Dendropanax trifidus Sap Suppresses Body Weight Increase in db/db, db/+, +/+ Mice of Both Sexes

The homozygous (db/db) alleles of BKS.Cg-*Dock7^m^* +/+ *Lepr^db^*/J lines, which have an obese phenotype, were used in this study. Female and male mice with genotypes of db/db, db/+, +/+ were administered daily with vehicle or DT sap over the course of 6 weeks and their body weights were measured weekly. Although we did not find a significant change in food or water consumption by DT sap administration (Supplementary Figure S1), DT sap-injected mice exhibited a suppression of body weight increase. This effect was clearly observed after 2–3 weeks of DTsap administration in all genotypes, and in both sexes, and was maintained until the end of the study period (Figure 1).

In detail, in female db/db, mice administered with vehicle control exhibited a steady rise in weight gain from week 9 to 15 which then plateaued till week 16, while mice injected with DT sap presented an increase only till week 11, and weight was stabilized thereafter (Figure 1A). Two-way ANOVA showed that there was a significant interaction in treatment x time (*p* < 0.001) and treatment (*p* < 0.001). In post hoc analysis, there were significant differences between the body weights of the vehicle group and the body weights of DT sap group throughout week 12~16 (post hoc Holm–Sidak, *p* = 0.006 at 12 week, *p* < 0.001 at 13~16 week, Figure 1A). In male db/db, body weights were also suppressed by the DT sap administration (Two-Way Anova, *p* < 0.001 for treatment, Figure 1B). The vehicle group exhibited a gradual increase in body weight till week 12 and was unchanged thereafter, while the DT sap group showed an increase till week 11 and a slight reduction since then, resulting in significant differences from week 12 to 16 (Holm–Sidak post hoc, *p* < 0.05 at 12, 13, 15 week, *p* < 0.01 at 14, 16 week, Figure 1B).

Suppression in body weight by DT sap was also observed in db/+ mice of both genders (two-way ANOVA, *p* < 0.001 for treatment, Figure 1C,D). Significant group differences in body weight were found from week 11 in female db/+ mice and from week 13 in male db/+ mice (Holm–Sidak post hoc, *p* = 0.047 at 11 week, *p* < 0.01 at 12–15 week, *p* < 0.001 at 16 week for female db/+, Figure 1C; *p* < 0.01 at 13, 14, 15 week, *p* = 0.012 at 16 week for male db/+, Figure 1D). In +/+ mice of both genders, body weight reduction was also observed (Two-Way Anova, for treatment: *p* = 0.021 for female +/+, Figure 1E; *p* < 0.001 for male +/+, Figure 1F). In female +/+ mice, no significant increase was found in post hoc analysis (Figure 1E), while there was a significant difference between groups from week 12 in male +/+ (Holm–Sidak post hoc, *p* = 0.012 at 12 week; *p* < 0.01 at 13, 14 week; *p* < 0.001 at 15, 16 week, Figure 1F). To summarize, compared to the vehicle control, DT sap administration exhibited a significant suppression against body weight increase in db/db, db/+, +/+ mice of both genders.

### 3.2. Glycolysis Stress Test in Skeletal Muscle Cells Treated with Dendropanax trifidus Sap

Muscle cells play the biggest roles in energy expenditure in the body [3,6]. If basal metabolic rate, which is variable between people due to genetic diversities [1,26,27], is low, the risk of body weight gain can rise [1,2]. We observed that DT sap administration suppressed the increase in body weight (Figure 1). In order to examine whether DT sap changes the energy expenditure of muscle cell, we investigated the changes in the glycolytic and mitochondrial function of C2C12 murine muscle cells treated with various concentrations of DTsap (Agilent Seahorse XF system, see Methods), (Figure 2 and Figure 3). The Agilent Seahorse XF energy map revealed contrasting metabolic signatures between DT sap-treated cells and vehicle-treated cells (Figure 2A). Vehicle-treated cells (blue) were located in the middle of the energy map, while DT sap-treated cells (red, light blue, purple) were located near the ‘Energetic’ region which shows active utilization of both metabolic pathways (i.e., mitochondrial respiration as well as glycolysis), suggesting a higher energy expenditure in the cells treated with DT sap (Figure 2A).

The Agilent Seahorse XF Glycolysis Stress Test was performed according to the manufacturer’s instructions. We observed that DT sap treatment significantly increased Glycolysis (Student’s *t*-test, *p* = 0.030, 0.027, 0.0079 for 62.5, 250, 500 μg/mL DT sap 1 h treatment; *p* = 0.0483, 0.0208 for 125, 250 μg/mL DT sap 24 h treatment, N = 3, Figure 2C). DT sap treatment significantly improved the glycolytic capacity (Student’s *t*-test, *p* = 0.028, 0.012, 0.013 for 62.5, 125, 250 μg/mL DT sap 1 hr treatment; *p* = 0.0072, 0.040 for 125, 250 μg/mL DT sap 24 h treatment, N = 3, Figure 2D). DT sap treatment exhibited significant increases in Glycolytic reserve (Student’s *t*-test, *p* = 0.040, 0.008 for 62.5, 125 μg/mL DT sap 1 h treatment; *p* = 0.041, 0.0032, 0.036 for 62.5, 125, 250 μg/mL DT sap 24 h treatment, N = 3, Figure 2E). Glycolytic reserve was significantly increased after 24 h in for the 62.5 and 125 ug/mL concentrations (Student’s *t*-test, *p* = 0.019, 0.029 for 62.5, 125 μg/mL DT sap 24 h treatment, N = 3, Figure 2F). In conclusion, DT sap increased glycolytic function in C2C12 myotubes.

### 3.3. Mitochondrial Stress Test in C2C12 Muscle Cells Treated with Dendropanax trifidus Sap

Next, to examine whether DT sap treatment changes mitochondrial respiration in the muscle, we performed Agilent Seahorse XF Mito Stress Test according to the manufacturer’s instructions. OCR was directly measured and major parameters of mitochondrial function (basal respiration, ATP production, maximal respiration, and spare respiratory capacity) were calculated (Figure 3A).

DT sap-treated cells exhibited a higher basal respiration (Student’s *t*-test, *p* = 0.024, 0.012, 0.012 for 62.5, 125, 250 μg/mL DT sap, N = 3, Figure 3B). ATP production was increased by DT sap treatment (Student’s *t*-test, *p* = 0.018, 0.011, 0.027 for 62.5, 125, 250 μg/mL DT sap, N = 3, Figure 3C). Maximal respiration was also increased by DT sap treatment (Student’s *t*-test, *p* = 0.043, 0.0014, 0.0084 for 62.5, 125, 250 μg/mL DT sap, N = 3, Figure 3D). Spare respiratory capacity was significantly increased by DT sap treatment (Student’s *t*-test, *p* = 0.0049, 0.0131 for 125, 250 μg/mL DT sap, N = 3, Figure 3E). The Mito Stress Test results showed that DT sap increases mitochondrial respiration in C2C12 muscle cells.

### 3.4. Estrogen Receptor Activation by Dendropanax trifidus Sap

**Figure 4:**
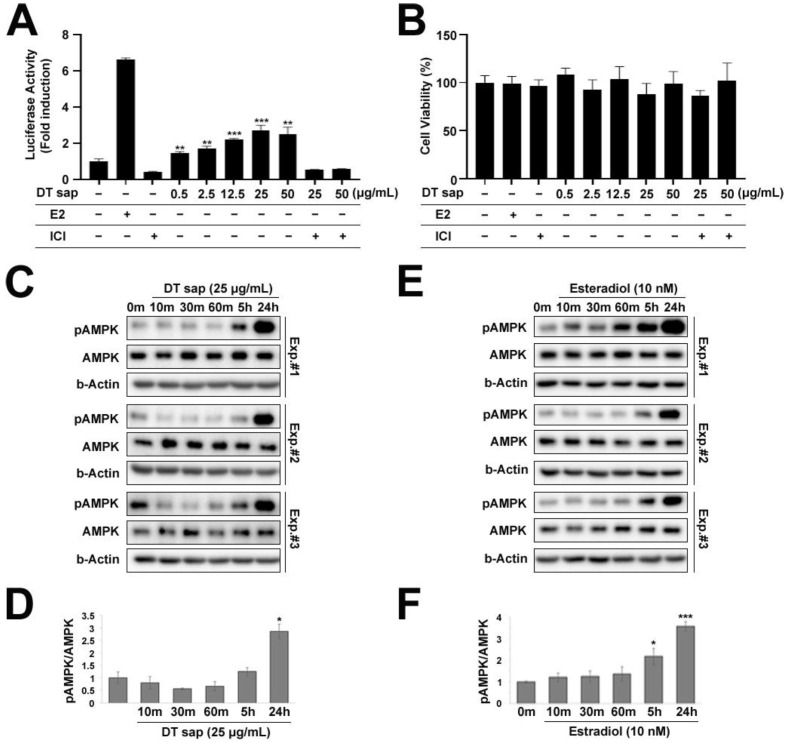
Estrogen receptor assay for *Dendropanax trifidus* (DT) sap and AMPK-mediated signaling. **(A**,**B**) “−” and “+” describe the absence or existence of the left-indicated reagent in the medium. (**A**) Estrogen-mediated luciferase reporter assay. hERα HeLa 9903 estrogen reporter cell line was used to measure estrogen activity in various concentrations (0, 0.5, 2.5, 12.5, 50 µg/mL) of DT sap. Estradiol (E2, 10 nM) was used as a positive control. Estrogen receptor antagonist ICI (1 µM) was used in a competition assay with 25 or 50 µg/mL of DT sap. N = 3. Student’s *t*-test; ** *p* < 0.01, *** *p* < 0.001, comparison with negative control. (**B**) Cell viability assay. (**C**,**E**) Western blot images of C2C12 muscle cells treated with 25 μg/mL DT sap (**C**) or 10 nM estradiol (**E**) for the indicated time points and probed with antibodies against pAMPK, AMPK, and b-Actin. Three different experimental blots (Experiment #1, #2, #3) are shown. (**D**,**F**) Quantification of pAMPK/AMPK ratios of (**C**) or (**E**). N = 3. Student’s *t*-test: * *p* < 0.05; *** *p* < 0.001, comparison with vehicle control. Bars indicate mean ± S.D. (**A**,**B**) or S.E.M. (**D**,**F**).

In our previous study, DT sap was compared with Acer Saccharum (AS) sap using liquid chromatograph-tandem mass spectrometer, and we observed that estradiol was abundantly contained in DT sap compared to AS sap [25]. We were interested in knowing if estrogen may be an active component in DT sap-mediated signaling, thus we utilized human ERα-HeLa-9903 cell lines which contain a luciferase reporter gene at downstream of an estrogen responsive promoter element. Activation of this receptor by estrogen can then be studied by measuring luciferase activity through chemiluminescence detection. When the reporter cell line was treated with DT sap of various concentrations (0, 0.5, 2.5, 12.5, 25, 50 μg/mL) over 48 h, luciferase activity was observed to be concentration-dependent with saturation at 25 μg/mL (Student’s *t*-test, *p* = 0.0078, 0.0035, 0.00008, 0.00056, 0.0033 for 0.5, 2.5, 12.5, 25, and 50 μg/mL, respectively, compared with negative control, N = 3, Figure 4A). This activity was significantly reduced by the estrogen receptor antagonist ICI, suggesting that its activation is via the estrogen receptor (Figure 4A). Estradiol (10 nM) alone was also used as a positive control, and showed a strong induction of luciferase activity. ICI alone did not induce luciferase activity. Cell viability was not damaged by any tested concentration of DT sap as well as other reagents (Figure 4B).

In C2C12 muscle cells, cellular metabolism including glycolysis and mitochondrial respiration was significantly increased by DT sap (Figure 2 and Figure 3). AMPK signaling is an important regulator for muscle metabolism [8]. Therefore, to test whether the metabolism changes observed in C2C12 by DTsap (Figure 2 and Figure 3) are related with AMPK signaling, we investigated AMPK signaling activation in C2C12 cells treated with DT sap over several time periods (0, 10, 30, 60 min, 5 h, 24 h) by western blot. Phosphorylation of AMPK was gradually increased, resulting in a significant increase at 24 h since the initial treatment (Student’s *t*-test, *p* = 0.020 at 24 h since the treatment, N = 3 independent experiments, Figure 4C,D). Like DT sap treatment, estradiol treatment also activated AMPK in C2C12 muscle cells, and exhibited the maximum activity at 24 h during the investigated period (24 h) (Student’s *t*-test, *p* = 0.036 for 0 vs. 5 h; *p* = 0.00028 for 0 vs. 24 h, N = 3, Figure 4E,F).

### 3.5. AMPK/ACC Pathway Was Activated in C2C12 Muscle Cells by Dendropanax trifidus Sap Treatment

**Figure 5:**
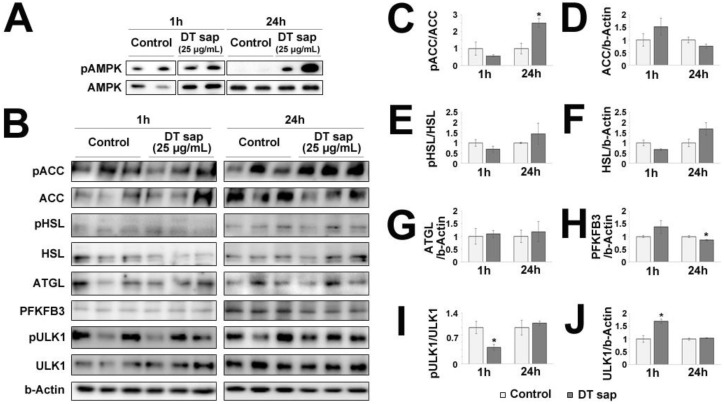
*Dendropanax trifidus* (DT) sap-induced signalings in C2C12 muscle cells. (**A**,**B**) Western blot images of C2C12 cells treated with vehicle control or 25 μg/mL DT for 1 h or 24 h and probed with pAMPK/AMPK antibodies (**A**) and AMPK-downstream signaling antibodies related with lipid metabolism (pACC, ACC, pHSL, HSL, ATGL); glucose metabolism (PFKFB3); autophagy and mitochondrial homeostasis (pULK1 and ULK1), and b-Actin (**B**). Three independent biological replicates are provided here. (**C**–**J**) Image analysis of western blot membranes of (**B**). Relative ratios for the indicated signals were calculated as follows: pACC/ACC (**C**); ACC/b-Actin (**D**); pHSL/HSL (**E**); HSL/b-Actin (**F**); ATGL/b-Actin (**G**); PFKFB3/b-Actin (**H**); pULK1/ULK1 (**I**); ULK1/b-Actin (**J**). 30 μg protein was loaded per lane. N = 3. Student’s *t*-test: * *p* < 0.05, comparison with vehicle control. Bars indicate mean ± S.E.M.

AMPK is a master regulator of cellular metabolism, which is located upstream of various cellular signaling pathways including lipid metabolism, glucose metabolism, autophagy and mitochondrial homeostasis [28]. To investigate further the downstream effects of DT sap on AMPK signaling, we performed western blots against major downstream signaling molecules at 1 h or 24 h after the initial treatment of DT sap. The following molecules were examined: phospho-ACC, ACC, phospho-HSL, HSL, and ATGL for lipid metabolism; PFKFB3 for glucose metabolism; phospho-ULK1 and ULK1 for autophagy and mitochondrial homeostasis (Figure 5). While DT sap did not induce phosphorylation of AMPK at 1 h, it significantly induced phosphorylation of AMPK at 24 h in C2C12 cells (Figure 4C,D and Figure 5A). Phospho-ACC/ACC was not altered at 1 h but significantly increased at 24 h after the treatment (Student’s *t*-test, *p* = 0.021 at 24 h since the treatment, N = 3 independent cultures, Figure 5B,C). There were no significant changes in ACC/b-Actin, phospho-HSL/HSL, HSL/b-Actin, ATGL/b-Actin at both time points (Figure 5B,D–G). PFKFB3/b-Actin was not altered at 1 h but significantly decreased at 24 h after the treatment (Student’s *t*-test, *p* = 0.048 at 24 h after the treatment, N = 3 independent cultures, Figure 5B,H). Moreover, phospho-ULK/ULK was significantly reduced at 1 h after the treatment but not after that (Student’s *t*-test, *p* = 0.043 at 1 h after the treatment, N = 3 independent cultures, Figure 5I). There was a significantly transient increase in ULK1/b-Actin by DT sap at 1 h (Figure 5J), explaining the reduced pULK/1ULK1 by DT sap at 1 h. The Western blot analysis indicates DT sap affects the molecular signaling related with AMPK-ACC within a 24 h time window as well as potential other components in muscle cells.

## 4. Discussion

In this work, we studied the effects of DT sap on body weight in vivo and muscle metabolism in vitro. Daily oral administration of DT sap over six weeks suppressed the increase of body weight in db/db, db/+, +/+ mice of both genders. In C2C12 muscle cells, DT sap treatment increased glycolysis and mitochondrial respiration and activated the AMPK/ACC signaling pathway which is potentially mediated by estrogen receptor alpha.

The antibody against phospho-AMPKα (Thr172) which we used in this study detects the phosphorylation of threonine 172 in both endogenous AMPKα1 and α2 isoforms of the catalytic subunit but not the regulatory beta or gamma subunits. This phosphorylation is required for AMPK activation [29,30,31]. Once the AMPK complex is activated, it phosphorylates major targets, activating or suppressing them, resulting in rewiring of cellular metabolism including lipid metabolism, glucose metabolism, autophagy, and mitochondrial homeostasis [28]. As skeletal muscle is a major contributor to glucose and lipid metabolism, it plays an essential role in whole-body energy expenditure. In skeletal muscle, exercise activates AMPK, and AMPK affects exercise capacity by regulating mitochondrial content, contraction-stimulated glucose uptake, and fatty acid metabolism [32]. In previous investigations using obese mice, AMPK activation also increased exercise capacity [33]. Acute exercise increases AMPK phosphorylation in human skeletal muscle in an intensity-dependent manner [34,35]. In this work, the dramatic increase in AMPK phosphorylation by DT sap treatment in murine muscle cells (Figure 4C,D) suggests enhancement of muscle cell metabolism (exercise-like effects) by DT sap and a potential association with the DT sap induced suppression of body weight observed here (Figure 1).

ACC is a major enzyme for biosynthesis and oxidation of fatty acids [9], and is a potential target of anti-obesity drugs [11,12]. In rodents, ACC1 (ACCα, 265 kDa) is expressed in lipogenic tissues which forms lipids, while ACC2 (ACCβ, 280 kDa) is the main isoform in oxidative tissue such as skeletal muscle [9,36]. In humans, ACC2 is the predominant isoform for both lipogenic and oxidative tissues [9,36]. Phosphorylation at Ser79 of ACC by AMPK inhibits the enzymatic activity of ACC [10]. The antibody used in this study specifically detects phosphorylation of ACC at Ser79. We observed here that DT sap treatment increased phosphorylation of both AMPK and ACC in mouse C2C12 muscle cells (Figure 5). Thus, as AMPK phosphorylates ACC and ACC phosphorylation suppresses fatty acid biosynthesis, this process may contribute to suppression of body weight increase (Figure 1).

While overexpression of PFKFB3 in mouse skeletal muscle promotes glycolysis [37], its expression is downregulated during myogenic cell differentiation [38]. Moreover, during differentiation, metabolic pathways such as glycolysis are inhibited [39]. Here, while there was no change in PFKFB3 level after 1 h of DT sap treatment, there was a significant reduction in its expression after 24 h compared to the control (Figure 5H). However, as glycolysis and mitochondrial respiration rate are increased by DT sap treatment (Figure 2 and Figure 3), the reduced expression of PFKFB3 by DT sap may indicate effects on myogenic cell differentiation rather than metabolism. ULK1 can be phosphorylated via both AMPK-dependent [40] and AMPK-independent pathways [41]. At 1 h of DT sap treatment, *p*-AMPK/AMPK was not altered (Figure 5A), while *p*-ULK1/ULK1 was significantly reduced due to increased ULK1 (Figure 5I,J), suggesting that enhanced *p*-ULK1/ULK1 here is via an AMPK-independent pathway.

In our previous report, we identified estrogen as one of the major components of DT sap [25]. In this study, we found that DT sap can signal through estrogen receptor alpha (Figure 4A). Estrogen receptor alpha is expressed in skeletal muscle and is critical for the regulation of metabolic homeostasis [42]. Its deletion in mouse increased adiposity caused by reductions in energy expenditure and exhibited glucose intolerance and insulin resistance [43,44,45]. In humans, women with the metabolic syndrome exhibit a reduced expression level of estrogen receptor alpha in muscle [46]. Therefore, at least partially, DT sap, which contains estrogen as a major component, might contribute to the control of body weight by modulating muscle metabolism via estrogen receptor alpha.

Taken together, our findings here allow us to hypothesize that DT sap increases energy expenditure in muscle cells, which may be via estrogen mediated AMPK signaling, thus contributing to the suppression of body weight increase. However, more studies will have to be performed to elucidate the molecular mechanisms by which this occurs. Possible approaches such as examining RNAi mediated regulation of the estrogen receptor under DT sap treatment, and also whether other compounds present in DT sap exhibit synergistic effects with estrogen on muscle cell energy expenditure and body weight reduction are expected for future study.

## Data Availability

The data presented in this study are available on request from the corresponding author.

## Supplementary Materials

The following supporting information can be downloaded at: https://www.mdpi.com/article/10.3390/nu14051098/s1. Figure S1: Average daily food or water consumption. Daily consumption of food (A) or water (B) was recorded and averaged after two weeks of daily oral administration of control vehicle or *Dendropanax trifidus* sap (0.2 mg/g body weight). N = 3 mice per group. Bars indicate mean ± S.E.M.
