# Supplementary material for: Dendropanax trifidus Sap-Mediated Suppression of Obese Mouse Body Weight and the Metabolic Changes Related with Estrogen Receptor Alpha and AMPK-ACC Pathways in Muscle Cells
Source: Nutrients. 2022 Mar 5;14(5):1098. doi: 10.3390/nu14051098 (PMC8912501; doi:10.3390/nu14051098)
Supplement: Supplementary file 1 [file nutrients-14-01098-s001.zip › nutrients-1622234-supplementary.pdf]

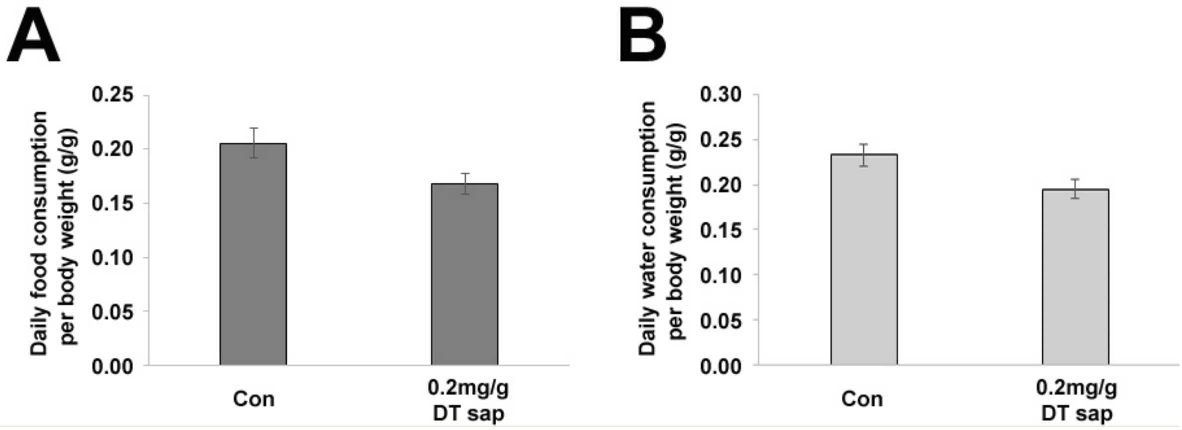

Figure S1. Average daily food or water consumption. Daily consumption of food (A) or water (B) was recorded and averaged after two weeks of daily oral administration of control vehicle or *Dendropanax Trifidus* sap (0.2 mg/g body weight). N = 3 mice per group. Bars indicate mean ± S.E.M.
